# Surgical treatment of apical prolapse Number 9 – 2026

**DOI:** 10.61622/rbgo/2026FPS9

**Published:** 2026-07-15

**Authors:** Marair Gracio Ferreira Sartori, Luiz Gustavo de Oliveira Brito, Lucas Schreiner, Aljerry Dias do Rego, Ana Selma Bertelli Picoloto, Andreisa Paiva Monteiro Bilhar, Carlos Del Roy, Daniela Siqueira Prado, Emerson de Oliveira, Jorge Milhem Haddad, Jussara Maria Valentim Cavalcante Nunes, Leticia Maria de Oliveira, Marilene Vale de Castro Monteiro, Rafael Mendes Moroni, Sergio Brazileiro Martins

**Affiliations:** 1 Escola Paulista de Medicina Universidade Federal de São Paulo São Paulo SP Brazil Escola Paulista de Medicina, Universidade Federal de São Paulo, São Paulo, SP, Brazil; 2 Faculdade de Medicina de Ribeirão Preto Universidade de São Paulo Ribeirão Preto SP Brazil Faculdade de Medicina de Ribeirão Preto, Universidade de São Paulo, Ribeirão Preto, SP, Brazil; 3 Pontifícia Universidade Católica do Rio Grande do Sul Porto Alegre RS Brazil Pontifícia Universidade Católica do Rio Grande do Sul, Porto Alegre, RS, Brazil; 4 Universidade Federal do Amapá Macapá AP Brazil Universidade Federal do Amapá, Macapá, AP, Brazil; 5 Universidade Federal do Rio Grande do Sul Porto Alegre RS Brazil Universidade Federal do Rio Grande do Sul, Porto Alegre, RS, Brazil; 6 Universidade Federal do Ceará Fortaleza CE Brazil Universidade Federal do Ceará, Fortaleza, CE, Brazil; 7 Centro Universitário Faculdade de Medicina do ABC Santo André SP Brazil Centro Universitário Faculdade de Medicina do ABC, Santo André, SP, Brazil; 8 Universidade Federal de Sergipe SE Brazil Universidade Federal de Sergipe, SE, Brazil; 9 Centro Universitário Faculdade de Medicina do ABC Santo André SP Brazil Centro Universitário Faculdade de Medicina do ABC, Santo André, SP, Brazil; 10 Hospital das Clínicas Faculdade de Medicina Universidade de São Paulo São Paulo SP Brazil Hospital das Clínicas, Faculdade de Medicina, Universidade de São Paulo, São Paulo, SP, Brazil; 11 Universidade Federal do Piaui Teresina PI Brazil Universidade Federal do Piaui, Teresina, PI, Brazil; 12 Escola Paulista de Medicina Universidade Federal de São Paulo São Paulo SP Brazil Escola Paulista de Medicina, Universidade Federal de São Paulo, São Paulo, SP, Brazil; 13 Universidade Federal de Minas Gerais Belo Horizonte MG Brazil Universidade Federal de Minas Gerais, Belo Horizonte, MG, Brazil; 14 Universidade Estadual do Oeste do Paraná Cascavel PR Brazil Universidade Estadual do Oeste do Paraná, Cascavel, PR, Brazil; 15 Escola Paulista de Medicina Universidade Federal de São Paulo São Paulo SP Brazil Escola Paulista de Medicina, Universidade Federal de São Paulo, São Paulo, SP, Brazil

## Abstract

Apical genital prolapse represents a failure of DeLancey’s level I support.

Isolated vaginal hysterectomy does not correct apical prolapse.

All surgery for uterine or vaginal vault prolapse should include apical suspension.

Sacrocolpopexy is the most durable technique for correcting vaginal vault prolapse, with the exception of colpocleisis.

Vaginal techniques remain valid options in selected patients.

Uterine preservation, when based on shared decision-making, is possible and safe for carefully evaluated patients.

Recognition of the apical component in advanced cystocele is essential to prevent recurrence.

Correction of the genital hiatus reduces the risk of recurrence, regardless of the surgical approach.

Intraoperative cystoscopy is mandatory in vaginal uterosacral suspensions.

Concomitant correction of stress urinary incontinence should be considered when diagnosed clinically or functionally.

## Recommendations

**Table:** 

Recommendations for the surgical treatment of apical genital prolapse	Level of evidence	Grade of recommendation
All surgery for uterine or vaginal vault prolapse should include an apical suspension technique, regardless of the surgical approach.	I	A
Isolated vaginal hysterectomy should not be performed as the sole treatment for apical genital prolapse.	I	A
Sacrocolpopexy should be considered the most durable technique for correcting vaginal vault prolapse, excluding colpocleisis.	I	A
Laparoscopic or robotic sacrocolpopexy offers similar efficacy and lower morbidity compared with the open approach.	I	A
Vaginal techniques using native tissue (uterosacral, sacrospinous, or iliococcygeal fixation) are valid alternatives for selected patients or those at higher surgical risk.	II	B
Intraoperative cystoscopy is mandatory in vaginal uterosacral suspensions to assess ureteral patency.	I	A
Uterine preservation can be safely offered for selected patients through shared decision-making.	II	B
Sacrohysteropexy is an effective alternative for women who desire uterine preservation, when performed by experienced surgeons.	II	B
Colpocleisis is an effective and safe option for patients with advanced apical prolapse, high surgical risk, and lack of desire for penetrative vaginal sexual activity.	I	A
Correction of the genital hiatus should be considered when enlarged, regardless of the surgical approach used.	II	B
Concomitant correction of stress urinary incontinence should be evaluated and discussed in surgical planning.	I	A
Recognition and correction of the apical component in advanced cystoceles significantly reduces the risk of recurrence.	I	A
The Manchester procedure may be considered in selected cases of uterine prolapse with predominant cervical elongation.	II	B
Anterior abdominal wall suspension techniques may be used in specific scenarios, although long-term evidence is lacking.	III	C

Grade of recommendation: A – strong, B – moderate, C – based on consensus/expert opinion. Level of evidence based on: I – systematic reviews, II – randomized clinical trials, III – observational studies

## Background

Pelvic organ prolapse (POP) is a highly prevalent condition with a significant impact on women’s quality of life, especially after menopause. It is estimated that up to 50% of multiparous women have some degree of prolapse and about 11% of them will require corrective surgery during their lifetime.^(1,2)^

Among the affected compartments, apical genital prolapse plays a central role, since inadequate support of the vaginal or uterine apex compromises the effectiveness and durability of corrections performed in the anterior and posterior compartments. Anatomical studies have shown that the apical support corresponds to DeLancey’s level I, formed mainly by the uterosacral and cardinal ligaments, the failure of which results in the descent of the uterus or vaginal vault.^(3,4)^

Current understanding of POP recognizes that advanced cystoceles and rectoceles rarely occur in isolation and are frequently associated with unrecognized or undertreated apical defects. Inadequate correction of the apical compartment is one of the main factors associated with surgical recurrence.^(5-7)^

Apical genital prolapse is defined as the descent of the apical compartment of the vagina towards the vaginal introitus, and may involve the uterus, the cervix in patients undergoing subtotal hysterectomy, or the vaginal vault after total hysterectomy, as well as the intestinal loops, characterizing enterocele.^(3)^ It is a prevalent condition, especially in multiparous and older adult women, with a significant impact on quality of life. Surgical treatment aims to restore apical support, relieve symptoms, prevent recurrence, and preserve vaginal function whenever possible. Several surgical techniques are described, with obliterative and reconstructive approaches via vaginal, open abdominal, laparoscopic, or robotic routes.

### What is the obliterative surgical technique for correcting apical prolapse?

Colpocleisis is the main obliterative surgery indicated for patients with advanced apical prolapse (stages III and IV), whether uterine or post-hysterectomy, who present with high surgical risk and no desire for penetrative vaginal intercourse. It can be performed with or without hysterectomy, provided that uterine pathology is ruled out if the uterus is preserved.^(1,2,4)^

Surgical technique summary: hydrodistension is performed with a vasoconstrictor solution, followed by excision of a rectangular flap of the anterior (AVW) and posterior (PVW) vaginal walls, maintaining a strip of vagina on each side. The suburethral area of the AVW is preserved for potential future sling surgery, and the endopelvic fascia is also preserved. The canal is closed using interrupted polyglactin sutures to approximate the vaginal walls, followed by perineorrhaphy to reduce the genital hiatus.^(5,6)^

Success rates range from 90% to 98%, with low perioperative morbidity and low recurrence rates.^(4,7-10)^ Serious complications are rare, and regret rates are minimal when the indication is appropriate.^(8-10)^ Vaginal hysterectomy is associated with longer operative time and greater blood loss.

### What are reconstructive techniques and which ones are available?

These are techniques that reconstruct the anatomy, preserving the vaginal canal and therefore penetrative vaginal sexual activity. There are several techniques that allow for adequate fixation of the apical compartment.^(11)^ These include:

- Sacrocolpopexy, sacrocervicopexy, sacrohysteropexy.
- Uterosacral vaginal fixation and uterosacral hysteropexy.
- Sacrospinous vaginal fixation and sacrospinous hysteropexy.
- Iliococcygeal colpopexy (vaginal fixation) and iliococcygeal hysteropexy.
- Anterior abdominal wall hysteropexy or colpopexy.
- Manchester procedure.

### Sacrocolpopexy, sacrocervicopexy, sacrohysteropexy: what are the technique, outcomes and complications?

Sacrocolpopexy is the suspension of the vaginal vault (in hysterectomized women) to the anterior longitudinal ligament of the sacrum, almost always using a mesh. Sacrocervicopexy (following a subtotal hysterectomy) and sacrohysteropexy (uterine preservation) are defined as the mesh suspension of the cervix to the anterior longitudinal ligament of the sacrum.^(11)^ These surgeries are indicated for the correction of symptomatic uterine or vaginal vault prolapse in stages II to IV, with or without prolapse of the AVW and PVW.^(12)^ Cervical preservation can reduce the incidence of mesh exposure.

Sacrocolpopexy is considered the gold standard for the treatment of vaginal vault prolapse, especially when greater durability and a lower risk of recurrence are desired.^(11-15)^ It consists of fixing the vaginal vault, cervix, or uterus to the anterior longitudinal ligament of the sacrum using a synthetic mesh.

The minimally invasive approach (laparoscopic or robotic) has similar efficacy to that of open surgery, with lower morbidity, less blood loss, and faster recovery.^(16-18)^ However, robotic surgery tends to have a longer operating time, but with advantages such as less blood loss and a lower conversion rate compared to laparoscopy, and may be especially useful in obese patients or those with multiple previous surgeries.

Sacrohysteropexy is an effective alternative for women who wish to preserve their uterus, presenting good anatomical and functional results when well indicated.^(14,16)^

#### Summary of the surgical technique

Assessment of the presacral space and feasibility of mesh fixation to the promontory. Opening of the retroperitoneum with identification of the right common iliac artery, left common iliac vein, right internal iliac artery and right ureter. Access through the right medial pararectal space towards the cervix/vaginal vault. Dissection of the presacral space with isolation of the superior hypogastric plexus from the anterior longitudinal ligament, avoiding the middle sacral vessels. Dissection of the rectovaginal space to the perineal body or the levator ani muscle, dissection of the vesicovaginal space to the level of the bladder neck. Supracervical hysterectomy when indicated or opening of the anterior and posterior broad ligament with bladder retraction. Fixation of a Y-shaped polypropylene mesh to the AVW and PVW using a long-lasting absorbable suture or to the uterine isthmus. Fixation of the mesh to the promontory with non-absorbable suture, followed by peritonealization.

Sacrocolpopexy provides superior results when compared to vaginal repairs with native tissue for the correction of apical prolapse.^(12,14)^

Anatomical success rates are high in short- and medium-term follow-ups, with reoperation rates below 5% in consolidated series, which confirms its durability and safety.^(12,14)^

Sacrohysteropexy results show significant improvement in symptoms and good anatomical correction. In a recent meta-analysis, sacrocolpopexy associated with hysterectomy demonstrated slightly higher rates of anatomical and subjective success compared to uterine preservation.^(13)^ However, when sacrohysteropexy is performed in well-selected patients, the results are considered satisfactory and similar in terms of symptomatic relief and functional improvement.^(16)^

Sacrocolpopexy carries a risk of potentially severe complications when compared to vaginal repairs. Among them are hemorrhage in the sacral region, bowel injuries, discitis or spondylodiscitis at the L5-S1 level, and mesh exposure or erosion. Among preventive strategies, the use of ultralight polypropylene meshes and fixation of the mesh to the vagina with long-lasting absorbable sutures stand out.^(12,14)^ Supracervical hysterectomy during abdominal sacrocolpopexy reduces the risk of mesh erosion compared to total hysterectomy.^(19)^ The association between hysterectomy and sacrocolpopexy does not increase the overall risk of mesh-related complications.^(16)^

## Uterosacral vaginal fixation and uterosacral hysteropexy: what are the techniques, outcomes and complications?

These aim to restore apical support of the uterus or vagina in symptomatic apical prolapses in stages I and II, anchoring them to the uterosacral ligaments at the time of hysterectomy, with or without the use of mesh. These procedures can be performed vaginally, laparoscopically, robotically or, less frequently, laparotomically.^(11)^ It is known as the High McCall procedure.^(6)^ In patients who wish to preserve the uterus, uterosacral hysteropexy may be indicated. Intraoperative cystoscopy is mandatory in vaginal suspensions due to the risk of ureteral obstruction, which occurs in 4%-5% of cases.^(20-23)^

### Summary of surgical technique

Identification of the uterosacral ligaments, followed by suturing of the ligaments using a non-absorbable suture (such as polyester) or a long-lasting absorbable suture (such as polyglactin) close to the ischial spine and the ipsilateral anterior and posterior vaginal wall (1 to 3 stitches). Alternatively, a McCall posterior culdoplasty suture can be performed: starting at the midline of the posterior vaginal wall, driving through the ipsilateral uterosacral ligament, running a transverse suture through the peritoneum to the contralateral uterosacral ligament and exteriorizing it back through the posterior vaginal mucosa. In the high approach, the ureters must be identified and retracted away from the suture site between the uterosacral ligament and the vaginal vault, following the retroperitoneal opening. In cases of uterine preservation, the cervix or uterine isthmus is fixed to the uterosacral ligaments.

The cure or satisfaction rate varies between 67% (stage III prolapse) and 92% (stage II prolapse).^(24)^ Recurrence after hysteropexy is generally low. In a large prospective cohort, the one-year apical anatomical recurrence was 7.5% for uterine preservation surgery, compared to 17.2% for vault suspension hysterectomy, with a lower adjusted relative risk for recurrence and failure in hysteropexy. Hysteropexy was also associated with shorter operative time, shorter hospital stay, less opioid use, and fewer procedural complications, with no differences in functional or quality of life outcomes between the groups.^(25)^ Short-term case series of extraperitoneal uterosacral hysteropexy report high rates of anatomical and subjective cure, low complication rates, and minimal vaginal length loss, although these data require confirmation in larger, long-term studies.^(20)^

Complications include hematomas, infection, and deep vein thrombosis. Ureteral obstruction requiring suture removal occurs in approximately 4%-5% of cases.^(22)^ In the medium and long term, recurrence of the prolapse may be observed.^(11,24)^ Urinary tract infection was found in 20% of postoperative uterosacral hysteropexy cases as the most prevalent adverse event.^(7)^ Neuropathic pain resulting from neural entrapment was reported by 1.6% of patients, manifesting as pain in the gluteal region or posterior thigh, in the S2-S4 distribution, and is generally resolved with medication or suture removal.^(22,23)^ Other complications include bladder injury (1%), pulmonary (2%-3%) and cardiac (<1%) complications, postoperative ileus (0.1%) and small bowel obstruction (0.8%).^(23)^ Anatomical recurrence of the prolapse, especially that related to cervical elongation, is more common after hysteropexy compared to hysterectomy-based suspension, resulting in higher reoperation rates for recurrence correction.^(6)^

## Sacrospinous vaginal fixation and sacrospinous hysteropexy: what are the techniques, outcomes and complications?

In these procedures, the vagina or uterus is anchored to the sacrospinous ligament using non-absorbable sutures or mesh. Sacrospinous vaginal fixation is indicated in patients with symptomatic vaginal vault prolapse when a transvaginal reconstructive approach is preferred.^(11)^ Sacrospinous hysteropexy is indicated in patients with symptomatic prolapse who wish to preserve the uterus, when reconstructive surgery is chosen.^(11)^

### Summary of surgical technique(26)

Vaginal infiltration with vasoconstrictor solution, followed by access to the sacrospinous ligament via either AVW or PVW. The anterior approach is indicated in concomitant cystocele; dissection begins at the pubocervical fascia towards the tendinous arch of the pelvic fascia and is directed posteriorly toward the ischial spine. The posterior approach is faster; following a midline vaginal incision, the pararectal fossa is dissected directly to the ischial spine. Access is achieved with or without the use of suture-capturing devices (such as silicone harpoon or Capio needle), allowing palpation and dissection of the sacrospinous ligament. When these devices are not used, retractors are necessary to pass the sutures manually. This involves placing either two distinct sutures or fixing a harpoon into the sacrospinous ligament, approximately 1.5 cm and 2.0 cm medial to the ischial spine. Suture placement can be bilateral (which is mandatory when mesh is used) or unilateral on the right side. The sutures, once anchored to the ligament, are passed through the vaginal apex or cervix. If mesh is used, it is secured to these ligamentous points bilaterally, fixed in the vagina or cervix, and followed by vaginal closure.

The initial success rate is high (92.5%), but recurrence increases over time.^(27)^ The risk of vaginal vault prolapse recurrence when using a transvaginal approach without mesh is higher than the risk of recurrence following sacrocolpopexy (11.3% vs. 7.9%). In the long term, overall recurrence can reach 33.3%.^(28,29)^ The success rate of hysterofixation is 77.0%, which is similar to that of surgeries involving uterine removal.^(27)^

Dyspareunia is described in 12.8% of patients who underwent sacrospinous fixation. Tissue injuries (bladder, ureter and intestine) are reported in 4.95% of cases; gastrointestinal complications (such as ileus and intestinal obstruction) occur in 1.33% of procedures. Surgical site infection can occur in 3.30% of cases, and hemorrhage is described in 0.95% of surgeries.^(27)^ In hysteropexy, complications are similar to those observed with sacrospinous vaginal fixation, but the smaller dissection area minimizes operative time and may reduce the risk of visceral injury.^(28)^

## Iliococcygeal vaginal fixation and iliococcygeal hysteropexy: what are the techniques, outcomes and complications?

These procedures involve the suspension of the vaginal vault or uterus, anchoring them with sutures to the fascia of the iliococcygeus muscle, also known as the prespinal fascia, which is located more caudally and medially to the ischial spine.^(29)^

These are performed vaginally and utilize absorbable or permanent sutures to secure the patient’s native tissue, without the use of synthetic mesh.^(30)^ The use of the iliococcygeus muscle fascia for uterine fixation lacks extensive evaluation in the literature. It is considered a technical variation of iliococcygeal colposuspension that incorporates uterine preservation.^(31)^ This technique is rarely performed in Brazil.

The main indications for vaginal fixation or iliococcygeal hysteropexy include women with apical prolapse, especially when seeking to avoid the use of mesh. It is also indicated for patients with contraindications to abdominal or laparoscopic surgery, as well as situations where maintaining vaginal length and sexual function is prioritized.

### Summary of surgical technique(32)

Opening of the posterior vaginal wall up to the fornix, or up to the reflection with the cervix, when present. Dissection of the rectovaginal space and identification of any enterocele hernial sac. In the presence of the uterus, adequate dissection and exposure of the posterior aspect of the cervix are necessary, where the suspension sutures will be anchored to the cervix. Opening and exposure of the pararectal fossa through the rectal pillar, unilaterally or bilaterally, using Breisky-Navratil long retractors. Unlike sacrospinous hysteropexy, in iliococcygeal hysteropexy it is not necessary to expose the coccygeous muscle-sacrospinous ligament complex, but only the fascia of the iliococcygeus muscle, which is located caudally and medially to the ischial spine. One or two sutures are passed through the iliococcygeus fascia, and this can be repeated on the contralateral side. Bilateral fixation is less suitable for cases of uterine suspension compared to vaginal vault suspension due to the difficulty of tissue apposition on both sides. The sutures are then transfixed through the cervical stroma at its most distal portion, or through the vaginal vault, ensuring they are buried during the closure of the posterior vaginal mucosa. When the sutures are tied, the cervix or vaginal vault is suspended from the iliococcygeal fascia. The suspension must be symmetrical in order to maintain a centered vaginal axis.

At a mean follow-up of five years, subjective cure rates of 88.6% and objective cure rates of 84.1% were reported.^(33)^ At the 10-year follow-up, overall success rates remained at 74.4%.^(34)^ Comparative studies indicate that, compared to abdominal sacrocolpopexy, iliococcygeal fixation has a shorter operative time (78 vs. 140 minutes), with similar anatomical results and lower complication rates.^(34)^

Another relevant point is the maintenance of sexual function. Most studies suggest that, by preserving vaginal length, the technique has a less negative impact on sexual satisfaction than sacrospinous fixation. However, some shortening may occur, especially in cases of advanced prolapse.^(31,35,36)^

Unlike vaginal vault suspension to iliococcygeus muscle, extensive literature is not available for iliococcygeal hysteropexy.^(33,37)^ In a single retrospective cohort study with a median follow-up of four years, success rates of 75.3% for stages I and II prolapse and 48% for stages III and IV prolapse were reported.^(37)^ The results of iliococcygeal colpopexy in association with hysterectomy are superior to those of hysteropexy in this technique.^(37)^

Among the most common complications are intraoperative bleeding, usually self-limiting, rectal wall injury, vascular laceration adjacent to the levator ani muscle^(38)^ and transient pelvic or gluteal pain, less prevalent than in sacrospinous fixation.

In the only available study on iliococcygeal hysteropexy, no intraoperative complications were reported.^(31)^ In contrast to sacrospinous suspension, this technique provides greater distance from clinically relevant neurovascular structures, normally located near the ischial spine and the coccygeal-sacrospinous ligament complex.^(29,38)^

Serious complications, such as vascular, ureteral, or neurological injuries, are extremely rare.^(31,35)^ Postoperatively, urinary tract infection is reported in approximately 8% of cases, persistent gluteal pain lasting more than four weeks in 1.4%, and lower limb neuropathy also in 1.4% of cases.^(31)^

### Anterior abdominal wall hysteropexy or colpopexy: what are the techniques, outcomes and complications?

Anterior abdominal wall hysteropexy or colpopexy techniques are surgical alternatives in selected clinical scenarios, particularly for young women who wish to preserve their uterus or when contraindications to the sacral approach exist. Despite promising initial outcomes, these techniques require further studies with greater methodological robustness to definitively establish their long-term efficacy and safety profiles.^(39-45)^

They are characterized by the suspension of the cervix, isthmus or vaginal vault and occasionally, the fibromuscular layer of the vaginal wall to the anterior abdominal wall, with or without the use of mesh. Fixation points may involve the round ligament, the pectineal ligament, or regions adjacent to the anterior superior iliac spine (ASIS).^(11)^

The main indications include symptomatic apical prolapse in women who wish to preserve the uterus, contraindications to hysterectomy, failure or recurrence following previous apical suspension surgeries, and scenarios where the sacral or sacrospinous approach is unfeasible or not preferred. It may also be considered in centers where sacrocolpopexy presents a greater technical challenge.

### Surgical technique summary

**Round ligament hysteropexy:** consists of passing sutures or mesh through the round ligament at its uterine insertion, followed by extraperitonealization and fixation to the fascia of the anterior abdominal wall. Autologous or synthetic rectus fascia grafts can be used; these are positioned bilaterally, with their medial ends introduced through the abdominal cavity to the posterior aspect of the uterus, superior to the insertion of the uterosacral ligaments.^(39,40)^ The technique can be performed unilaterally, bilaterally, or via plicature of the round ligaments.

**Pectineal ligament suspension:** performed via an open abdominal or laparoscopic approach, the retropubic space is accessed to isolate the pectineal ligament. A non-absorbable mesh is fixed to the anterior aspect of the cervix, and its tails passed bilaterally through the pectineal ligament, promoting uterine elevation and stabilization.^(41,42)^

**Hysteropexy near the ASIS:** following dissection of the vesicovaginal space, a synthetic mesh is fixed to the anterior surface of the cervix and the fibromuscular layer of the anterior vaginal wall. The mesh arms are exteriorized bilaterally through incisions located 4 cm posterior to the ASIS and 2 cm superior to the iliac crest, and are then fixed symmetrically to the anterior abdominal wall. Closure of the peritoneum over the mesh is optional, and the technique can be performed via open or laparoscopic approaches.^(42-44)^

Although the available data are mostly observational, studies report high rates of anatomical and functional success, especially in young women with uterine preservation. Long-term follow-up studies describe recurrence rates of less than 1% and high satisfaction rates,^(45)^ in addition to satisfactory anatomical results in 87% of cases after five years.^(44)^

Pectineal ligament suspension showed a recurrence rate of 5.1% after a mean follow-up of 6.5 years, with maintenance of reproductive viability.^(41)^ Compared to sacrocervicopexy, hysteropexy of the anterior abdominal wall demonstrated less blood loss, shorter operative time, and lower recurrence rates.^(40)^ Unilateral fixation utilizing the round ligament resulted in complete reduction of the prolapse in 76.4% of cases and partial reduction in 21.8%.^(39)^

In lateral mesh suspension techniques, anatomical success rates of 88% in the anterior compartment, 86% in the apical compartment, and 81% in the posterior compartment were described, with 92.3% satisfaction after a mean follow-up of 7.5 years.^(42)^ Comparative studies have demonstrated better anatomical outcomes, greater satisfaction, and a lower rate of mesh exposure in uterine-sparing procedures compared to those associated with hysterectomy.^(43)^

Serious intraoperative complications are rare. The most frequently reported complications include pelvic pain or dyspareunia (up to 0.36%), new-onset stress urinary incontinence (12%), mesh exposure or erosion (0.9% to 6.5%, significantly lower in uterine-sparing procedures), and prolapse recurrence, ranging from 0.7% to 12%, depending on the technique and follow-up time.^(40-45)^ Available reports suggest reproductive safety for future pregnancies after pectineal ligament suspension.^(41)^

## Manchester operation: what is the technique, outcomes and complications?

This is surgery for uterine prolapse that includes anterior and posterior colporrhaphy, amputation of the cervix and fixation of the cardinal ligaments to the anterior surface of the cervical stump.^(46)^ The Manchester procedure is indicated for women with uterine prolapse associated with cervical elongation (endocervical canal length greater than 5 cm) who wish to preserve uterine and/or reproductive function or who present with significant comorbidities.

### Summary of surgical technique(46)

Anterolateral incision of the cervix down to the subepithelial plane, followed by a midline opening of the anterior vagina wall extending from the cervical incision to approximately 3 cm from the urethral meatus. Bladder dissection is performed, separating it from the cervix up to the urethrovesical junction and, laterally, close to the ischiopubic rami bilaterally. The cardinal-uterosacral ligament complex is clamped, sectioned, ligated and shortened bilaterally with polyglactin sutures. Cervical amputation is then performed. Anterior vaginal wall prolapse is corrected by repairing the pubocervical fascia. Subsequently, the cardinal and uterosacral ligaments are fixed to the anterior surface of the cervix with polyglactin suture, promoting uterine suspension toward the sacral concavity. The excess mucosa of the anterior vaginal wall is resected, followed by suturing, and the amputated cervical stump is re-epithelialized using Sturmdorf sutures. The procedure is completed with the closure of the anterior vaginal wall, posterior colporrhaphy, and perineorrhaphy (perineal body repair).

Compared to vaginal hysterectomy, the Manchester procedure did not demonstrate a significant difference in patient satisfaction, showing a prolapse cure rate of 81% and overall satisfaction of 89%. The group that underwent hysterectomy presented a higher rate of serious complications (1.9% versus 0.2%; p < 0.001), as well as longer operative time, greater perioperative blood loss, and longer hospital stay.^(47)^

In a series of patients with a mean age of 48.7 years, the Manchester procedure demonstrated an anatomical success rate of 97% and a patient satisfaction rate of 94.8%.^(48)^ When compared to sacrospinous hysteropexy, the Manchester procedure demonstrated a superior anatomical success rate (87.3% versus 77%).^(49,50)^

Complications associated with the Manchester procedure are generally infrequent and of low severity. The most frequently described include urinary tract infection (11.5%), hematoma (0.9%), cervical stenosis (0.5%), intraoperative bleeding (0.5%), and local infection (0.5%).^(49)^

## How to differentiate apical prolapse from other compartments?

Apical prolapse is identified by a detailed gynecological examination, with the aid of a vaginal speculum, allowing visualization of the cervix, vaginal vault, or cul-de-sac. Caudal descent of these structures during the Valsalva maneuver characterizes apical compromise.^(6)^

### When to indicate the vaginal, abdominal, laparoscopic, or robotic approach?

The choice of surgical approach should be individualized, considering the type of prolapse, age, comorbidities, previous surgeries, the surgeon’s experience, and available resources.

- Vaginal approach: indicated in older adult, frail patients or those at high surgical risk. Techniques such as sacrospinous or uterosacral fixation show good results.
- Open abdominal approach: currently reserved for specific cases, such as multiple previous abdominal surgeries or the need for associated abdominal procedures, due to higher morbidity.^(17,18)^
- Laparoscopic approach: replicates the principles of open sacrocolpopexy with lower morbidity, faster recovery, and high durability. It is the preferred approach in many centers.^(12-14)^
- Robotic approach: presents similar efficacy to that of laparoscopic approach, facilitating intracorporeal suturing, but with longer operative time and higher cost.^(17,18)^

Minimally invasive abdominal sacrocolpopexy is generally considered the most durable technique for vaginal vault prolapse.

## From what stage of cystocele should an associated apical prolapse be suspected?

Cystoceles beyond stage II usually present with an associated apical component. Descent of the vaginal apex can pull on the anterior vaginal wall, simulating or exacerbating the cystocele. Failure to recognize this apical component is associated with a higher risk of recurrence when only the anterior vaginal wall is corrected.^(51)^

## Does isolated vaginal hysterectomy treat apical prolapse?

No. Isolated vaginal hysterectomy does not correct the underlying apical support defect and is associated with a higher risk of subsequent vaginal vault prolapse. All hysterectomies performed for prolapse should be accompanied by an apical suspension technique, such as uterosacral or sacrospinous ligament fixation.^(12)^

## Which technique should be indicated when concomitant correction of urinary incontinence is necessary?

In patients with overt or occult stress urinary incontinence, concomitant correction with apical suspension is recommended, usually with a transobturator or retropubic sling, reducing the risk of persistence or the emergence of new symptoms. The decision should be individualized and shared with the patient.

## Should the genital hiatus be corrected concomitantly, regardless of the surgical approach?

Yes. In case of an enlarged genital hiatus, particularly with measurements greater than 4 cm, concomitant correction is recommended regardless of the surgical approach due to its association with a higher risk of prolapse recurrence. In these cases, posterior colpoplasty combined with perineorrhaphy is indicated.^(52)^

## When should cystoscopy be performed?

Cystoscopy is mandatory in surgeries for vaginal or uterine fixation to the uterosacral ligaments. It should be performed immediately after suturing to confirm bilateral ureteral patency. In the absence of a urinary stream, the suture should be repositioned.^(11)^

## What are the main postoperative care measures?

- Maintain relative rest during the first few days following surgery.
- Avoid lifting heavy objects (>5 kg) for six to eight weeks.
- Avoid strenuous activities that increase abdominal pressure.
- Optimize bowel function to prevent straining.
- Avoid sexual intercourse for approximately eight weeks.
- Observe for signs of infection, abnormal bleeding, or urinary changes.
- Strictly follow medical instructions regarding prescribed medications and follow-up.

## Final considerations

Apical genital prolapse requires an accurate diagnosis and tailored surgical correction to optimize quality of life and minimize recurrence rates. The choice of technique should be individualized, based on scientific evidence, patient characteristics, surgeon experience, and institutional resources. Ultimately, achieving robust apical suspension remains the primary determinant of long-term surgical success.

## References

[1] Buchsbaum GM, Lee TG (2017). Vaginal obliterative procedures for pelvic organ prolapse: a systematic review. Obstet Gynecol Surv.

[2] Grzybowska ME, Futyma K, Kusiak A, Wydra DG (2022). Colpocleisis as an obliterative surgery for pelvic organ prolapse: is it still a viable option in the twenty-first century? Narrative review. Int Urogynecol J.

[3] (2017). Practice Bulletin No. 176: pelvic organ prolapse. Obstet Gynecol.

[4] Hill AJ, Walters MD, Unger CA (2016). Perioperative adverse events associated with colpocleisis for uterovaginal and posthysterectomy vaginal vault prolapse. Am J Obstet Gynecol.

[5] Mueller MG, Ellimootil C, Abernethy MG, Mueller ER, Hohmann S, Kenton K (2015). Colpocleisis: a safe, minimally invasive option for pelvic organ prolapse. Female Pelvic Med Reconstr Surg.

[6] Federação Brasileira das Associações de Ginecologia e Obstetrícia (2025). Prolapso dos órgãos pélvicos. Femina.

[7] Zebede S, Smith AL, Plowright LN, Hegde A, Aguilar VC, Davila GW (2013). Obliterative LeFort colpocleisis in a large group of elderly women. Obstet Gynecol.

[8] FitzGerald MP, Richter HE, Siddique S, Thompson P, Zyczynski H, Weber Ann, Pelvic Floor Disorders Network (2006). Colpocleisis: a review. Int Urogynecol J Pelvic Floor Dysfunct.

[9] Wheeler TL, Richter HE, Burgio KL, Redden DT, Chen CC, Goode PS (2005). Regret, satisfaction, and symptom improvement: analysis of the impact of partial colpocleisis for the management of severe pelvic organ prolapse. Am J Obstet Gynecol.

[10] Crisp CC, Book NM, Cunkelman JA, Tieu AL, Pauls RN, Society of Gynecologic Surgeons' Fellows' Pelvic Research Network (2016). Body image, regret, and satisfaction 24 weeks after colpocleisis: a multicenter study. Female Pelvic Med Reconstr Surg.

[11] (2020). Joint Report on Terminology for Surgical Procedures to Treat Pelvic Organ Prolapse. Female Pelvic Med Reconstr Surg.

[12] Matthews CA (2016). Minimally invasive sacrocolpopexy: how to avoid short- and long-term complications. Curr Urol Rep.

[13] Maher C, Yeung E, Haya N, Christmann-Schmid C, Mowat A, Chen Z (2023). Surgery for women with apical vaginal prolapse. Cochrane Database Syst Rev.

[14] Gluck O, Blaganje M, Veit-Rubin N, Phillips C, Deprest J, O'Reilly B (2020). Laparoscopic sacrocolpopexy: a comprehensive literature review on current practice. Eur J Obstet Gynecol Reprod Biol.

[15] Moroni RM, Juliato CR, Cosson M, Giraudet G, Brito LG (2018). Does sacrocolpopexy present heterogeneity in its surgical technique? A systematic review. Neurourol Urodyn.

[16] Tius V, Arcieri M, Taliento C, Pellecchia G, Capobianco G, Simoncini T (2025). Laparoscopic sacrocolpopexy with concurrent hysterectomy or uterine preservation: a metanalysis and systematic review. Int J Gynaecol Obstet.

[17] Chang CL, Chen CH, Chang SJ (2022). Comparing the outcomes and effectiveness of robotic-assisted sacrocolpopexy and laparoscopic sacrocolpopexy in the treatment of pelvic organ prolapse. Int Urogynecol J.

[18] Chang CL, Chen CH, Yang SS, Chang SJ (2022). An updated systematic review and network meta-analysis comparing open, laparoscopic and robotic-assisted sacrocolpopexy. J Robot Surg.

[19] Nassif J, Yadav GS, Orejuela FJ, Turrentine MA (2022). Rate of mesh erosion after sacrocolpopexy with concurrent supracervical compared with total hysterectomy: a systematic review and meta-analysis. Obstet Gynecol.

[20] Ossin D, Ramirez-Caban L, Hurtado E (2020). Extraperitoneal uterosacral ligament hysteropexy: a novel treatment for apical compartment prolapse. Urology.

[21] Unger CA, Walters MD, Ridgeway B, Jelovsek JE, Barber MD, Paraiso MF (2015). Incidence of adverse events after uterosacral colpopexy for uterovaginal and posthysterectomy vault prolapse. Am J Obstet Gynecol.

[22] Flynn MK, Weidner AC, Amundsen CL (2006). Sensory nerve injury after uterosacral ligament suspension. Am J Obstet Gynecol.

[23] Chung CP, Kuehl TJ, Larsen WI, Yandell PM, Shull BL (2012). Recognition and management of nerve entrapment pain after uterosacral ligament suspension. Obstet Gynecol.

[24] Margulies RU, Rogers MA, Morgan DM (2010). Outcomes of transvaginal uterosacral ligament suspension: systematic review and metaanalysis. Am J Obstet Gynecol.

[25] Brennand EA, Scime NV, Huang B, Edwards AD, Kim-Fine S, Hall J (2025). Hysterectomy versus uterine preservation for pelvic organ prolapse surgery: a prospective cohort study. Am J Obstet Gynecol.

[26] Declas E, Giraudet G, Delplanque S, Rubod C, Cosson M (2020). How we perform a posterior sacrospinous ligament fixation by the vaginal route. Int Urogynecol J.

[27] McDonald J, Salehi O, Sathianathen N, Dowling C, Elmer S (2025). Sacrospinous fixation versus uterosacral ligament suspension in managing apical prolapse. World J Urol.

[28] Zhang W, Cheon WC, Zhang L, Wang X, Wei Y, Lyu C (2022). Comparison of the effectiveness of sacrospinous ligament fixation and sacrocolpopexy: a meta-analysis. Int Urogynecol J.

[29] Maher CF, Murray CJ, Carey MP, Dwyer PL, Ugoni AM (2001). Iliococcygeus or sacrospinous fixation for vaginal vault prolapse. Obstet Gynecol.

[30] Suh DH, Jeon MJ (2018). Risk factors for the failure of iliococcygeus suspension for uterine prolapse. Eur J Obstet Gynecol Reprod Biol.

[31] Krissi H, Stanton SL (2005). Bilateral iliococcygeal fixation for vaginal vault prolapse and enterocele repair using a new suturing device--the digital needle driver. BJOG.

[32] Shull BL, Capen CV, Riggs MW, Kuehl TJ (1993). Bilateral attachment of the vaginal cuff to iliococcygeus fascia: an effective method of cuff suspension. Am J Obstet Gynecol.

[33] Serati M, Braga A, Bogani G, Maggiore UL, Sorice P, Ghezzi F (2015). Iliococcygeus fixation for the treatment of apical vaginal prolapse: efficacy and safety at 5 years of follow-up. Int Urogynecol J.

[34] Serati M, Salvatore S, Athanasiou S, Scancarello C, Ghezzi F, Braga A (2021). Ten years' follow-up after iliococcygeus fixation for the treatment of apical vaginal prolapse. Int Urogynecol J.

[35] Milani R, Cesana MC, Spelzini F, Sicuri M, Manodoro S, Fruscio R (2014). Iliococcygeus fixation or abdominal sacral colpopexy for the treatment of vaginal vault prolapse: a retrospective cohort study. Int Urogynecol J.

[36] Olsen AL, Smith VJ, Bergstrom JO, Colling JC, Clark AL (1997). Epidemiology of surgically managed pelvic organ prolapse and urinary incontinence. Obstet Gynecol.

[37] Braga A, Amato G, Caccia G, Papadia A, Treglia G, Scancarello C (2024). Iliococcygeus fixation for the treatment of vaginal vault prolapse: a systematic review and meta-analysis. Minerva Urol Nephrol.

[38] Paz-Levy D, Yohay D, Neymeyer J, Hizkiyahu R, Weintraub AY (2017). Native tissue repair for central compartment prolapse: a narrative review. Int Urogynecol J.

[39] Hsieh CH (2011). A new laparoscopic technique for uterine prolapse: one-sided uterine fixation through the round ligament. Int Urogynecol J.

[40] Li S, Ji M, Zhao Z (2015). The effectiveness of two different laparoscopic surgeries for apical support of pelvic organ prolapse. Eur J Obstet Gynecol Reprod Biol.

[41] Joshi VM, Otiv SR, Dagade VB, Borse M, Majumder RN, Shrivastava M (2015). Pectineal ligament hysteropexy for uterine prolapse in premenopausal women by open and laparoscopic approach in Indian urban and rural centers. Female Pelvic Med Reconstr Surg.

[42] Veit-Rubin N, Dubuisson JB, Lange S, Eperon I, Dubuisson J (2016). Uterus-preserving laparoscopic lateral suspension with mesh for pelvic organ prolapse: a patient-centred outcome report and video of a continuous series of 245 patients. Int Urogynecol J.

[43] Veit-Rubin N, Dubuisson J, Constantin F, Lange S, Eperon I, Gomel V (2019). Uterus preservation is superior to hysterectomy when performing laparoscopic lateral suspension with mesh. Int Urogynecol J.

[44] Rimailho J, Talbot C, Bernard JD, Hoff J, Bécue J (1993). L'hystéropexie antéro-latérale par voie abdominale. Résultats et indications. A propos d'une série de quatre-vingt-douze patients. Ann Chir.

[45] Khanam RA, Rubaiyat A, Azam MS (2014). Sling for correcting uterine prolapse: twelve years experience. Mymensingh Med J.

[46] Skiadas CC, Goldstein DP, Laufer MR (2006). The Manchester-Fothergill procedure as a fertility sparing alternative for pelvic organ prolapse in young women. J Pediatr Adolesc Gynecol.

[47] Bergman I, Söderberg MW, Kjaeldgaard A, Ek M (2017). Cervical amputation versus vaginal hysterectomy: a population-based register study. Int Urogynecol J.

[48] Liger Guerra C, Sabonet Morente L, Hidalgo Fernandez JM, Navarro Romero M, Gonzalez CE, Jimenez-Lopez JS (2025). The Manchester procedure as a uterine-preserving alternative for uterine prolapse due to cervical elongation: a short- and mid-term clinical analysis. Medicina (Kaunas).

[49] Enklaar RA, Schulten SF, van Eijndhoven HW, Weemhoff M, van Leijsen SA, van der Weide MC (2023). Manchester procedure vs sacrospinous hysteropexy for treatment of uterine descent: a randomized clinical trial. JAMA.

[50] Güler Çekiç S, Aktoz F, Urman B, Aydin S (2024). A systematic review of uterine cervical elongation and meta-analysis of Manchester repair. Eur J Obstet Gynecol Reprod Biol.

[51] Delancey JO (2012). Surgery for cystocele III: do all cystoceles involve apical descent?: observations on cause and effect. Int Urogynecol J.

[52] Handa VL, Blomquist JL, Carroll MK, Muñoz A (2021). Genital hiatus size and the development of prolapse among parous women. Female Pelvic Med Reconstr Surg.

